# Correlation of Speckle-Tracking Echocardiography with Traditional Biomarkers in Predicting Cardiotoxicity among Pediatric Hemato-Oncology Patients: A Comprehensive Evaluation of Anthracycline Dosages and Treatment Protocols

**DOI:** 10.3390/children10091479

**Published:** 2023-08-30

**Authors:** Andrada Mara Ardelean, Ioana Cristina Olariu, Raluca Isac, Ruxandra Jurac, Cristiana Stolojanu, Mircea Murariu, Ana-Olivia Toma, Laurentiu Braescu, Adelina Mavrea, Gabriela Doros

**Affiliations:** 1 Department of Pediatrics, “Victor Babes” University of Medicine and Pharmacy, Eftimie Murgu Square 2, 300041 Timisoara, Romania; andrada.micsescu-olah@umft.ro (A.M.A.); olariu.cristina@umft.ro (I.C.O.); isac.raluca@umft.ro (R.I.); steflea.ruxandra@umft.ro (R.J.); gdoros@gmail.com (G.D.); 2 Doctoral School, “Victor Babes” University of Medicine and Pharmacy, Eftimie Murgu Square 2, 300041 Timisoara, Romania; stolojanu.cristiana@umft.ro (C.S.); mircea.1192@yahoo.com (M.M.); braescu.laurentiu@umft.ro (L.B.); 3 Louis Turcanu Emergency Hospital for Children, Iosif Nemoianu Street 2, 300011 Timisoara, Romania; 4 Department of Dermatology, “Victor Babes” University of Medicine and Pharmacy, Eftimie Murgu Square 2, 300041 Timisoara, Romania; toma.olivia@umft.ro; 5 Department of Cardiovascular Surgery, “Victor Babes” University of Medicine and Pharmacy, Eftimie Murgu Square 2, 300041 Timisoara, Romania; 6 Center for Translational Research and Systems Medicine (CERT-MEDS), “Victor Babes” University of Medicine and Pharmacy, Eftimie Murgu Square 2, 300041 Timisoara, Romania; 7 Department of Internal Medicine I, Cardiology Clinic, “Victor Babes” University of Medicine and Pharmacy Timisoara, Eftimie Murgu Square 2, 300041 Timisoara, Romania

**Keywords:** echocardiography, cardiotoxicity, hematology, oncology, pediatrics

## Abstract

Speckle tracking-echocardiography (STE) is a novel non-invasive imaging tool capable of quantifying myocardial deformation, and thus holds promise in detecting early subclinical myocardial injury. This study aimed to evaluate the correlation of STE with traditional biomarkers in predicting anthracycline-induced cardiotoxicity in the context of varying dosages and treatment protocols in pediatric hemato-oncology patients. We conducted a retrospective study involving pediatric hemato-oncology patients undergoing anthracycline-based chemotherapy. A total of 99 patients were included in the final analysis, with 82 receiving Doxorubicin, of which 58.5% were males, and 17 receiving Epirubicin, of which 70.6% were males, with a median of 10 years old. Traditional biomarkers, such as Troponin I (cTnI) and B-type natriuretic peptide (BNP), were compared with STE parameters, including the global longitudinal strain (GLS), Simpson method of discs (SMOD), and myocardial performance index (MPI). A comprehensive evaluation was conducted based on different dosages of anthracyclines and different treatment protocols, with a follow-up period of one year post-chemotherapy. It was observed that the cTnI levels in the Doxorubicin group were significantly higher (3.2 ng/mL, *p* = 0.002) than in the Epirubicin group (2.7 ng/mL). However, BNP and NT-proBNP levels were not significantly different between the two groups (*p* = 0.096 and *p* = 0.172, respectively). Regarding STE parameters, a significant negative correlation was observed between the anthracycline dose and GLS (Rho = −0.411, *p* = 0.001), indicating increased cardiotoxicity with dose elevation. The SMOD and MPI gave significantly better values in the Epirubicin group (59.2 and 0.41 vs. 54.4 and 0.36, respectively). However, the ROC analysis did not find GLS, SMOD, or MPI to be significant independent predictors of cardiotoxicity (*p* > 0.05). There was also considerable variation in cardiotoxicity between the Doxorubicin and Epirubicin study groups, suggesting that the risk of cardiotoxicity is not solely determined by dose. Our study underlines the potential of STE as a sensitive tool for the early detection and prediction of anthracycline-induced cardiotoxicity in pediatric hemato-oncology patients, but only in association with the clinical findings and cardiac biomarkers. While traditional biomarkers still play a role, STE can offer a more accurate prediction of cardiac risk, potentially leading to better management and outcomes for these patients.

## 1. Introduction

The advancements in pediatric oncology treatments over recent years have significantly increased survival rates, which is a testament to the progress of medical science [1]. However, these treatment protocols often involve the administration of chemotherapy drugs such as anthracyclines, which, while highly effective, are also known to induce cardiotoxicity [2]. This side effect has become a growing concern as it can lead to substantial long-term health complications, particularly in pediatric patients who have a potentially long life ahead post-treatment [3,4].

Cardiotoxicity induced by anthracyclines is often irreversible and may progress to symptomatic heart failure, a risk that underscores the necessity for early detection [5]. Traditional methods of assessing cardiotoxicity, including echocardiography, electrocardiography (ECG), nuclear imaging, and various cardiac biomarkers, have been instrumental in this regard [6,7]. Nonetheless, these methods sometimes lack sensitivity, making it challenging to detect early cardiac dysfunction before irreversible damage occurs.

In recent years, speckle-tracking echocardiography (STE) has emerged as a novel technique that offers superior sensitivity and precision in detecting subtle myocardial deformations, including global longitudinal strain (GLS) [8]. Previous studies in adults have demonstrated that reductions in GLS can be an early marker of anthracycline-induced cardiotoxicity [9]. However, the utility of STE in the pediatric population, particularly in hematology-oncology patients, is less well-established and warrants further investigation.

The influence of chemotherapy protocols, including anthracycline dosage and combination with other drugs such as cytarabine, etoposide, and cyclophosphamide, on the risk of cardiotoxicity is an equally pertinent area of research [10,11]. A comprehensive evaluation of these factors, in conjunction with cardiac monitoring using both traditional methods and STE, could offer valuable insights into the optimization of treatment protocols to minimize cardiotoxicity while preserving antineoplastic efficacy [12]. Moreover, the impact of individual patient factors such as age, sex, body surface area, and specific disease type on cardiotoxicity risk is not yet fully understood [13]. This understanding is crucial for personalizing treatment protocols and implementing appropriate cardiac monitoring strategies, thus reducing the long-term cardiac morbidity associated with pediatric cancer treatment.

It is hypothesized that speckle-tracking echocardiography is correlated with the traditional biomarkers in predicting cardiotoxicity and that this correlation will be influenced by anthracycline dosages and treatment protocols. Therefore, the main objective of the current study is to determine the predictive role of STE parameters for anthracycline-induced cardiotoxicity in pediatric hemato-oncology patients. The secondary objectives are to determine the correlation between the STE findings and traditional cardiac biomarkers and to observe the differences between different anthracycline types. Lastly, it was planned to observe the differences between patients treated with Doxorubicin and Epirubicin.

## 2. Materials and Methods

### 2.1. Design and Ethics

This study was designed as a retrospective cohort study conducted using medical records of pediatric patients admitted to the oncology ward of the “Louis Turcanu” Emergency Children’s Hospital affiliated with the “Victor Babes” University of Medicine and Pharmacy between 2019 and 2022. Ethical approval was obtained from the institutional review board, and all procedures were carried out following the Declaration of Helsinki and local data protection guidelines. Informed consent was obtained from all individual participants or their parents/legal guardians.

### 2.2. Inclusion and Exclusion Criteria

**Figure 1:**
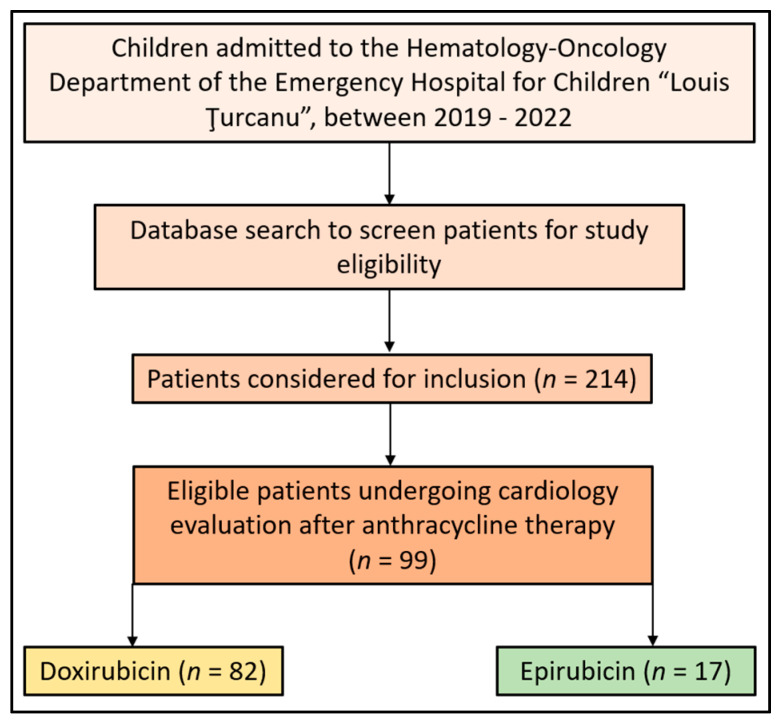
Patient flowchart.

Patients newly diagnosed with cancer requiring anthracycline therapy during the study period were included. Baseline values were established at their first presentation, and patients were subsequently monitored following the oncology protocol or upon the development of cardiac symptomatology. The specific time points for data collection were baseline (before therapy), immediately post-chemotherapy, and at the one-year follow-up. Patients who survived until the one-year follow-up were included in the analysis. Patients were excluded if they had previously received oncological treatment before baseline establishment, had a known cardiac pathology, or had started treatment at our hospital but decided to continue elsewhere. The patient flowchart is described in Figure 1.

The variables considered for evaluation in this study comprised age, age range, body mass index (BMI), BMI percentile categories, gender, cancer histology, chemotherapy treatment protocol, laboratory markers (cardiac troponins, brain natriuretic peptide, and creatine kinase), and cardiac measurements (ejection fraction, global longitudinal strain, Simpson’s method of discs, myocardial performance index, and electrocardiogram findings).

Before the initiation of chemotherapy, baseline laboratory values and echocardiographic (echo) parameters were collected. These served as a reference for subsequent comparative analyses. Immediately following the chemotherapy regimen, a comprehensive set of laboratory values and echo variables were recorded. It is pertinent to note that these post-chemotherapy measurements were integral to our analysis. They were used as primary predictors in our model to forecast the risk of cardiotoxicity within the one-year follow-up period. At the end of one year post-chemotherapy, a final assessment was made to identify any instances of cardiotoxicity. This long-term follow-up was crucial to validate the predictions made based on the immediate post-chemotherapy measurements.

### 2.3. Materials Used and Definitions

The diagnosis of cardiac toxicity was made by identifying ultrasound alterations within one year after chemotherapy. Cardiotoxicity was defined by an LVEF absolute decrease of more than 10% and a final LVEF less than 50%. As a secondary analysis, a relative GLS decrease of more than 15% was considered a single measure of probable cardiotoxicity if the LVEF decline was more than 10% but the final LVEF was greater than 50% or if there was a relative GLS decrease of more than 15% [14,15,16]. The investigations were carried out using a GE VIVID E9 echocardiograph, obtaining good quality images with ECG signals in apical view (3, 4, and 2 chambers). Speckles, myocardial reflectors, were tracked throughout the cardiac cycle, with changes in speckle position used to determine myocardial strain. Cardiac function was also measured using traditional methods, such as M-mode dimension ejection fraction Teicholtz, volume ejection fraction Simpson (SMOD) [17], and tissue Doppler imaging, to measure the myocardial performance index (MPI) [18]. Any other abnormalities appearing during treatment were documented and observed. Data were collected from the patients’ paper and digital records, as well as direct measurements during investigations.

The GLS frame rate was set at 40 fps, with a 50 Hz probe. Decreases in GLS (values less negative or closer to 0) may indicate decreased cardiac function and potentially cardiac injury [19]. The normal range for SMOD is generally considered to be between 55% and 70%, while values below 55% are usually considered indicative of reduced systolic function. Normal MPI values are generally considered to be less than 0.40, and these may vary slightly depending on whether they are measured by tissue Doppler imaging or flow Doppler imaging. Values above 0.40 indicate worsening global cardiac function. The Epirubicin dosage was converted to Doxorubicin dosage using a conversion factor of 0.67 [20].

### 2.4. Statistical Analysis

Data were analyzed using the IBM SPSS v.26 statistical software package. Descriptive statistics, including means, standard deviations, and percentages, were used to summarize the data. The differences between groups were evaluated using the independent t-test for continuous variables and the Chi-square or Fisher’s exact test for categorical variables, as appropriate. Pearson’s correlation was used to determine the associations between continuous variables, measured by the “rho” coefficient. Two separate endpoints were defined: (1) LVEF absolute decrease >10% and final LVEF <50% for the primary outcome, and (2) relative GLS decrease >15% for the secondary outcome. Kaplan–Meier analysis was employed to assess the risk of cardiotoxicity by treatment type. Receiver operating characteristic (ROC) curves were constructed to assess the predictive accuracy of GLS and other traditional cardiac biomarkers for cardiotoxicity. A multivariate logistic regression was performed to determine the predictors associated with cardiotoxicity. All tests were two-tailed, and *p*-values less than 0.05 were considered statistically significant.

## 3. Results

### 3.1. Background Data of Patients

The background data of the study participants are thoroughly examined in Table 1. The participant group consisted of 82 patients who had been treated with Doxorubicin and 17 patients treated with Epirubicin. The mean age of the Doxorubicin group was 10.7 ± 4.4 years, while the Epirubicin group had a slightly lower mean age of 10.2 ± 3.6 years. The difference in age was not statistically significant (*p* = 0.471), implying the two groups were similar in age distribution.

The analysis of the Body Mass Index (BMI) showed that the mean BMI was 20.5 ± 4.6 kg/m^2^ for the Doxorubicin group and 21.3 ± 5.8 kg/m^2^ for the Epirubicin group. The BMI between the two groups was not statistically significantly different (*p* = 0.308). When analyzing the BMI percentile categories, no significant difference was found between the two groups (*p* = 0.748). In the Doxorubicin group, 3.7% of patients were in the >85% category, 14.6% were in the 50–85% category, 37.8% were in the 15–50% category, 36.6% were in the 5–15% category, and 7.3% were in the <5% category. In the Epirubicin group, these proportions were 5.9%, 23.5%, 35.3%, 23.5%, and 11.8%, respectively. In terms of gender distribution, the Doxorubicin group comprised 58.5% males and 41.5% females, while in the Epirubicin group, 70.6% were males and 29.4% were females. No significant difference was found in the gender distribution between the two groups (*p* = 0.354).

### 3.2. Oncological Data

The cohort included a total of 99 patients, with the largest proportion diagnosed with B-cell acute lymphoblastic leukemia (B-ALL) (29.3%). Patients with T-cell acute lymphoblastic leukemia (T-ALL), Hodgkin lymphoma, nephroblastoma, and acute myeloid leukemia (AML) each made up 12.1% of the cohort. The less prevalent cancers were non-Hodgkin lymphoma (7.1%), osteosarcoma (6.1%), rhabdomyosarcoma (5.1%), and medulloblastoma (2.0%).

The incidence of cardiac toxicity was not uniformly distributed across the different cancer types and treatment protocols. It ranged from 0% in medulloblastoma patients who underwent the MET-HIT2000 protocol to 60% in patients with rhabdomyosarcoma treated with the CWS protocol. The second highest rate of cardiac toxicity was observed in osteosarcoma patients treated with the EURAMOS protocol (50.0%), followed by T-ALL and Hodgkin lymphoma patients who experienced cardiac toxicity rates of 35.7% and 33.3%, respectively. For B-ALL patients, who made up the largest percentage of the cohort, the incidence of cardiac toxicity was 24.2%, whereas for non-Hodgkin lymphoma and AML patients, the rates were 28.5% and 16.7%, respectively. Nephroblastoma patients also had a cardiac toxicity rate of 33.3%, as presented in Table 2.

### 3.3. Cancer Function Assessment

Cardiac troponin I (cTnI) and troponin T (cTnT) remain sensitive and specific biomarkers of myocardial injury. According to Table 3, the mean cTnI level in the Doxorubicin group was 3.2 ± 0.5 ng/mL, significantly higher than in the Epirubicin group (2.7 ± 0.9 ng/mL), with a *p*-value of 0.002, suggesting more myocardial injury in the Doxorubicin group. Similar results were found for cTnT, with mean levels of 1.5 ± 0.6 ng/mL in the Doxorubicin group and 0.8 ± 0.5 ng/mL in the Epirubicin group. This difference was also statistically significant, with a *p*-value of less than 0.001.

Brain natriuretic peptide (BNP) and N-terminal pro-brain natriuretic peptide (NT-proBNP) serve as markers of myocardial strain and heart failure. The Doxorubicin group had slightly higher mean BNP and NT-proBNP levels (260 ± 94 pg/mL and 282 ± 77 pg/mL, respectively) compared to the Epirubicin group (220 ± 61 pg/mL and 255 ± 53 pg/mL, respectively). However, these differences were not statistically significant (*p* = 0.096 and *p* = 0.172, respectively), indicating that both anthracyclines exert similar effects on myocardial strain. Creatine kinase (CK) and its isoforms are additional biomarkers of myocardial injury. The mean CK-MB level remained higher in the Doxorubicin group (33 ± 10 U/L) compared to the Epirubicin group (29 ± 8 U/L), although this difference was not statistically significant (*p* = 0.129). Conversely, the mean CK level (referring to the total CK level) was significantly higher in the Doxorubicin group (270 ± 91 U/L) compared to the Epirubicin group (204 ± 68 U/L), with a *p*-value of 0.006. These data suggest more extensive myocardial injury in the Doxorubicin group.

The ejection fraction (EF), which is an indicator of cardiac systolic function, showed a mean value of 62.1 ± 5.5% in the Doxorubicin group and 64.7 ± 5.9% in the Epirubicin group. The difference in the mean EF was not statistically significant (*p* = 0.087). However, when categorized, a significant difference was noted (*p* = 0.031). There were more patients with EF > 70% in the Epirubicin group (29.4%) than in the Doxorubicin group (7.3%). The mean Global Longitudinal Strain (GLS), an indicator of cardiac contractility, was −15.5 ± 4.6 in the Doxorubicin group and −18.3 ± 5.8 in the Epirubicin group, demonstrating a significantly better GLS in the Epirubicin group (*p* = 0.034), as presented in Table 4.

**Figure 2:**
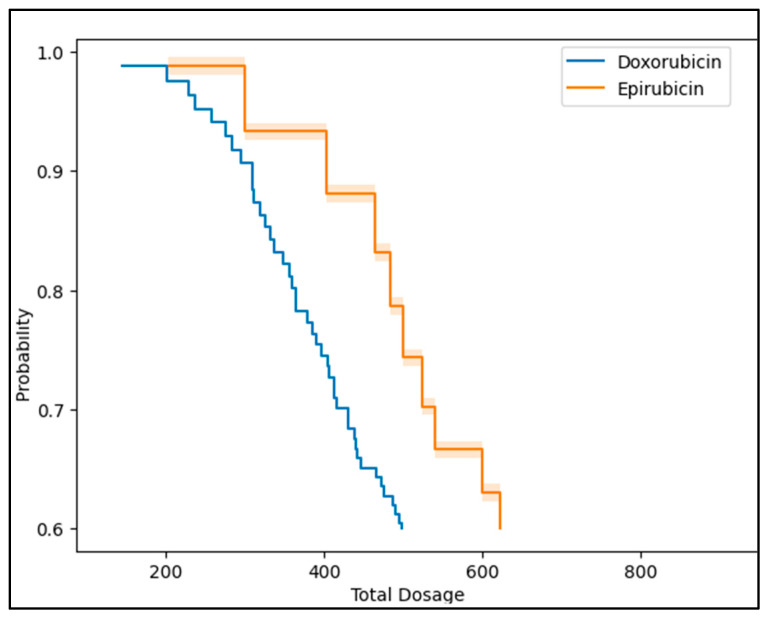
Kaplan–Meier analysis of cardiotoxicity risk by treatment type.

The Simpson method of discs (SMOD) and myocardial performance index (MPI) showed mean values of 54.4 ± 5.8 and 0.36 ± 0.05, respectively, in the Doxorubicin group and 59.2 ± 6.6 and 0.41 ± 0.07, respectively, in the Epirubicin group. These differences were statistically significant, indicating better cardiac function in the Epirubicin group (*p* = 0.003 for SMOD and *p* = 0.001 for MPI). Regarding the Electrocardiogram (ECG) findings, 64.6% of the Doxorubicin group and 76.5% of the Epirubicin group had normal findings, whereas abnormal findings were observed in 35.4% and 23.5% of patients, respectively. This difference, however, was not statistically significant (*p* = 0.346). Cardiac ultrasound results were normal in 43.9% of the Doxorubicin group and 64.7% of the Epirubicin group, with abnormal findings in 56.1% and 35.3%, respectively. This difference was not statistically significant (*p* = 0.117). In terms of cardiotoxicity, the incidence was higher in the Doxorubicin group (32.9%) than in the Epirubicin group (17.6%), but the difference was not statistically significant (*p* = 0.212). Nevertheless, the Kaplan–Meier analysis of cardiotoxicity risk by treatment type, described in Figure 2, indicated that cardiotoxicity occurs at significantly lower doses for Doxorubicin compared to Epirubicin.

### 3.4. Correlation Analysis and Predictive Factors

**Figure 3:**
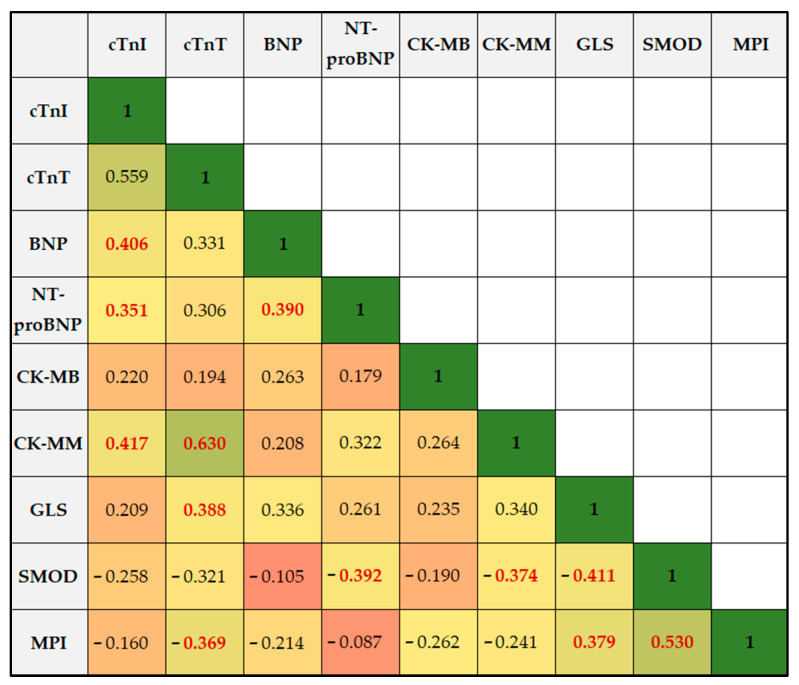
Correlation analysis of factors associated with cardiac toxicity.

A correlation analysis was performed and is described in Table 5 and Figure 3. The cardiac troponin I (cTnI) showed a statistically significant positive correlation with cardiac troponin T (cTnT) (Rho = 0.559, *p* = 0.001) and creatine kinase-MM (CK-MM) (Rho = 0.417, *p* = 0.014). However, cTnI did not exhibit a significant correlation with brain natriuretic peptide (BNP), NT-proBNP, or creatine kinase-MB (CK-MB). The cTnT demonstrated a statistically significant positive correlation with CK-MM (Rho = 0.630, *p* = 0.001) and the global longitudinal strain (GLS) (Rho = 0.388, *p* = 0.001), but it did not correlate significantly with BNP, NT-proBNP, or CK-MB.

BNP showed a significant positive correlation with NT-proBNP (Rho = 0.390, *p* = 0.001) but had no significant correlation with CK-MB or CK-MM. Moreover, the NT-proBNP correlated significantly negatively with the Simpson method of discs (SMOD) (Rho = −0.392, *p* = 0.004), but not with CK-MB or CK-MM.

**Figure 4:**
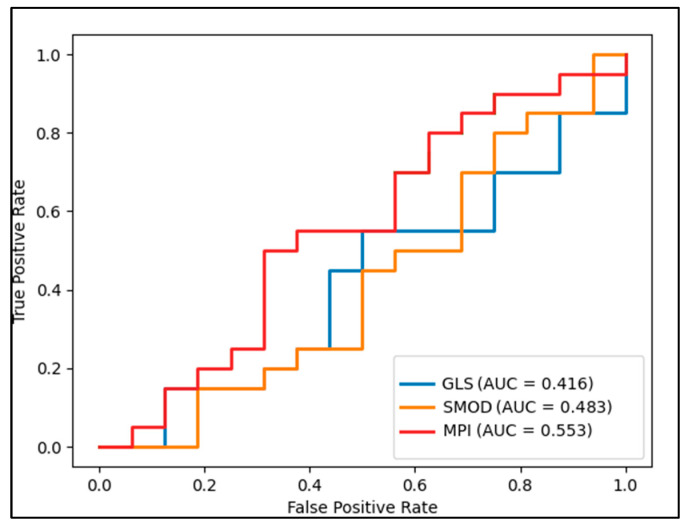
ROC analysis.

Regarding the speckle cardiac parameters, GLS showed a significant positive correlation with MPI (Rho = 0.379, *p* = 0.020) and a significant negative correlation with SMOD (Rho = −0.411, *p* = 0.001). Moreover, SMOD showed a significant negative correlation with GLS and a significant positive correlation with MPI (Rho = 0.530, *p* = 0.001), as well as with CK-MM (Rho = −0.374, *p* = 0.001). Although there were significant correlations identified between the cardiac measurement parameters and cardiac markers associated with cardiotoxicity after chemotherapy, the ROC analysis indicated a poor predictive value and did not show statistically significant results for GLS, SMOD, and MPI as independent predictors (Figure 4).

### 3.5. Regression Analysis

Table 6 presents the results of the regression analysis for the associations between various cardiac function measurements, biomarkers, and the development of cardiac toxicity. SMOD showed a strong association, with an odds ratio of 4.05, signifying a quadrupling in the odds of cardiac toxicity for each unit increase in the SMOD measure (*p* < 0.001). The MPI demonstrated a moderate association, with an odds ratio of 2.49 (*p* = 0.030). LVEF had an odds ratio of 3.16, implying a little over a three-fold increase in the odds of cardiac toxicity for each unit increase in LVEF, with a significant *p*-value (*p* < 0.001).

In terms of cardiac biomarkers, cTnI and cTnT showed statistically significant odds ratios of 1.41 (*p* = 0.042) and 3.91 (*p* < 0.001), respectively. The NT-proBNP and CK-MM levels showed a statistically significant association with cardiac toxicity, with respective odds ratios of 2.15 (*p* < 0.001) and 2.58 (*p* < 0.001). CK-MB, however, did not show a statistically significant association.

## 4. Discussion

### 4.1. Literature Findings

The study’s results indicate several key findings in the application of speckle-tracking echocardiography (STE) and Global Longitudinal Strain (GLS) in predicting cardiotoxicity among pediatric hemato-oncology patients. Predominantly, the study revealed statistically significant differences between the Doxorubicin and Epirubicin groups across multiple parameters, signifying variations in their respective cardiotoxicity profiles.

Our research highlighted that the level of myocardial injury markers, namely cardiac troponin I (cTnI) and troponin T (cTnT), were notably higher in the Doxorubicin group. Similar findings were reported by Lipshultz et al. [21], who found significantly elevated troponin levels in pediatric patients treated with anthracyclines, suggesting that these biomarkers might be early indicators of chemotherapy-related cardiotoxicity. The correlation between higher cTnI and cTnT levels with the Doxorubicin group mirrors their findings and could suggest a higher risk of myocardial damage in this group.

The current study demonstrated that both Doxorubicin and Epirubicin exhibited comparable effects on myocardial strain, as indicated by the B-type Natriuretic Peptide (BNP) and N-terminal pro B-type Natriuretic Peptide (NT-proBNP) levels. This aligns with the research of Horacek et al. [22], who reported that BNP levels increased in pediatric cancer patients after anthracycline treatment, reflecting cardiac strain. Despite the non-significant differences between our two groups, these biomarkers nonetheless remain indicative of myocardial stress in both treatment regimens.

It was further discovered that the creatine kinase-MM (CK-MM) level was significantly higher in the Doxorubicin group, suggesting more extensive myocardial injury in this group. Elevated CK-MM levels were previously associated with anthracycline-induced cardiotoxicity by Armenian et al. [23], signifying that this marker may be valuable in early cardiotoxicity detection. Our findings supplement this proposition, with the notable disparity between the two groups emphasizing the potential differential risk associated with the two drugs.

We found a significantly better GLS in the Epirubicin group, which is an important finding given the demonstrated prognostic value of GLS in earlier studies. This was echoed in a study by Thavendiranathan et al. [24], which found that GLS predicted early left ventricular dysfunction in adult patients undergoing chemotherapy. A better GLS in the Epirubicin group could suggest a lower likelihood of contractility impairment in these patients compared to those in the Doxorubicin group.

In addition, our study revealed statistically significant differences in the Simpson method of discs (SMOD) and myocardial performance index (MPI) between the two groups. Previous studies, such as the one conducted by Eidem et al. [25], have confirmed the relevance of these indices in monitoring cardiac function in pediatric cancer patients. The better cardiac function observed in the Epirubicin group further underscores the differential impact of the two treatment regimens on cardiac health. Interestingly, our study found that GLS and SMOD correlated significantly negatively, while GLS and MPI had a significant positive correlation. This emphasizes the importance of considering a comprehensive set of indices for a more accurate cardiotoxicity assessment. However, these STE parameters did not show significant results in the ROC analysis as independent predictors of cardiotoxicity, indicating the need for a multifaceted assessment approach including both STE parameters and traditional biomarkers.

Therefore, the current study sheds light on the utility of STE and its correlation with traditional cardiac biomarkers in predicting anthracycline-induced cardiotoxicity among pediatric hemato-oncology patients. Importantly, our findings provide crucial evidence-based insights for optimizing treatment protocols and cardiac monitoring strategies.

### 4.2. Study Limitations

Despite the potential merits of this study, several limitations must be acknowledged. The use of a retrospective cohort design potentially introduces selection bias and limits control over possible confounding variables. Additionally, we relied on medical records for data, which can result in missing or inaccurate data. The study also involves a single center, which could impact the generalizability of our findings to different clinical settings. Furthermore, the variety of cancer types and chemotherapy protocols might introduce heterogeneity into the cohort, potentially confounding the relationship between chemotherapy and cardiotoxicity. The reliance on GLS as a predictive marker for cardiotoxicity, while promising, is still a developing field; hence, the cut-off values used may not be universally applicable. Lastly, our exclusion of patients with pre-existing cardiac conditions may result in an underestimation of the true incidence of cardiotoxicity as these patients may be at a higher risk. These limitations should be considered when interpreting the findings of this study and developing future research strategies.

## 5. Conclusions

Doxorubicin-treated patients exhibited inferior cardiac function compared to those treated with Epirubicin, as suggested by poorer GLS and other echocardiographic measures such as SMOD and MPI. However, the ROC analysis did not support these echocardiographic indices as successful predictors of anthracycline-induced cardiotoxicity. Nonetheless, we identified significant correlations between these ultrasound parameters and cardiac biomarkers. Cardiotoxicity also seemed to occur at significantly lower dosages of Doxorubicin compared to Epirubicin. Therefore, despite the values of STE and GLS being non-invasive measures of cardiac function during chemotherapy, our findings indicate that they are useful only as supplements to traditional biomarkers and ongoing cardiac monitoring to detect and manage cardiotoxicity in this patient group.

## Figures and Tables

**Table 1:** Background data of study participants.

Variables	Doxorubicin (*n* = 82)	Epirubicin (*n* = 17)	*p*-Value
Age (mean ± SD)	10.7 ± 4.4	10.2 ± 3.6	0.471
Age range	1–18	1–17	–
BMI, kg/m^2^ (mean ± SD)	20.5 ± 4.6	21.3 ± 5.8	0.308
BMI percentile categories			0.748
>85%	3 (3.7%)	1 (5.9%)	
50–85%	12 (14.6%)	4 (23.5%)	
15–50%	31 (37.8%)	6 (35.3%)	
5–15%	30 (36.6%)	4 (23.5%)	
<5%	6 (7.3%)	2 (11.8%)	
Gender (*n*,%)			0.354
Male	48 (58.5%)	12 (70.6%)	
Female	34 (41.5%)	5 (29.4%)	
Dosage (mean ± SD)	323.6 ± 75.7	355.9 ± 92.0	0.126

BMI—body mass index; SD—standard deviation. The Epirubicin dosage was converted to Doxorubicin dosage using the conversion factor of 0.67 [20].

**Table 2:** Cancer histology and treatment protocol.

Cancer Histology	*n* (%)	Treatment Scheme	Cardiac Toxicity * (*n*,%)
Hodgkin lymphoma	12 (12.1%)	ABVD	4 (33.3%)
Non-Hodgkin lymphoma	7 (7.1%)	RCHOP	2 (28.5%)
Nephroblastoma	12 (12.1%)	ISPO	4 (33.3%)
Osteosarcoma	6 (6.1%)	EURAMOS	3 (50.0%)
Rhabdomyosarcoma	5 (5.1%)	CWS	3 (60.0%)
Medulloblastoma	2 (2.0%)	MET-HIT2000	0 (0.0%)
T-ALL	14 (14.1%)	ALL BFM 2014	5 (35.7%)
B-ALL	29 (29.3%)	ALL BFM 2014	7 (24.2%)
AML	12 (12.1%)	AML BFM 2014	2 (16.7%)

*—Proportion calculated of each cancer type. T-ALL—T-cell acute lymphoblastic leukemia; B-ALL—B-cell acute lymphoblastic leukemia; AML—acute myeloid leukemia; BFM—Berlin-Frankfurt-Münster; ABVD—Adriamycin (Doxorubicin), Bleomycin, Vinblastine, and Dacarbazine; RCHOP—Rituximab, Cyclophosphamide, Hydroxydaunorubicin (Doxorubicin), Oncovin (Vincristine), and Prednisone; EURAMOS—European and American Osteosarcoma Study Group; CWS (for rhabdomyosarcoma)—Cooperative Weichteilsarkom Studiengruppe; ISPO (for nephroblastoma)—International Society of Pediatric Oncology; MET-HIT—Multicenter Therapy Optimizing Study for Treatment of Children and Adolescents With Intracranial Medulloblastoma.

**Table 3:** Laboratory findings based on Anthracycline type.

Variables (Mean ± SD)	Normal Range *	Doxorubicin (*n* = 82)	Epirubicin (*n* = 17)	*p*-Value
cTnI	<2.0 ng/mL	3.2 ± 0.5	2.7 ± 0.9	0.002
cTnT	<1.4 ng/mL	1.5 ± 0.6	0.8 ± 0.5	<0.001
BNP	<200 pg/mL	260 ± 94	220 ± 61	0.096
NT-proBNP	<242 pg/mL	282 ± 77	255 ± 53	0.172
CK-MB	<25 U/L	33 ± 10	29 ± 8	0.129
CK	<200 U/L	270 ± 91	204 ± 68	0.006

* age adjusted values; SD—standard deviation; cTn—cardiac troponins; BNP—brain natriuretic peptide, CK—creatine kinase.

**Table 4:** Cardiac findings based on Anthracycline type.

Variables (Mean ± SD)	Doxorubicin (*n* = 82)	Epirubicin (*n* = 17)	*p*-Value
Initial EF (mean ± SD)	62.1 ± 5.5	64.7 ± 5.9	0.087
EF categories (initial)			0.031
50–60%	14 (17.1%)	2 (11.8%)	
60–70%	62 (75.6%)	10 (58.8%)	
>70%	6 (7.3%)	5 (29.4%)	
GLS (mean ± SD)	−15.5 ± 4.6	−18.3 ± 5.8	0.034
SMOD (mean ± SD)	54.4 ± 5.8	59.2 ± 6.6	0.003
MPI (mean ± SD)	0.36 ± 0.05	0.41 ± 0.07	0.001
ECG			
Normal findings	53 (64.6%)	13 (76.5%)	0.346
Abnormal	29 (35.4%)	4 (23.5%)	
Cardiac ultrasound			0.117
Normal findings	36 (43.9%)	11 (64.7%)	
Abnormal	46 (56.1%)	6 (35.3%)	
Cardiotoxicity (*n*,%)	27 (32.9%)	3 (17.6%)	0.212

SD—standard deviation; GLS—Global longitudinal strain; SMOD—Simpson method of discs; MPI—myocardial performance index; ECG—Electrocardiogram; EF—ejection fraction.

**Table 5:** Correlation analysis of the studied variables.

		cTnI	cTnT	BNP	NT-proBNP	CK-MB	CK-MM	GLS	SMOD	MPI
cTnI	Rho	1								
	*p*-value	-								
cTnT	Rho	0.559 **	1							
	*p*-value	0.001	-							
BNP	Rho	0.406 **	0.331	1						
	*p*-value	0.003	0.078	-						
NT-proBNP	Rho	0.351	0.306	0.390 **	1					
	*p*-value	0.040	0.192	0.001	-					
CK-MB	Rho	0.220	0.194	0.263	0.179	1				
	*p*-value	0.389	0.450	0.105	0.306	-				
CK-MM	Rho	0.417 *	0.630 **	0.208	0.322	0.264	1			
	*p*-value	0.014	0.001	0.250	0.146	0.131	-			
GLS	Rho	0.209	0.388 **	0.336	0.261	0.235	0.340	1		
	*p*-value	0.087	0.001	0.078	0.204	0.319	0.401	-		
SMOD	Rho	−0.258	−0.321	−0.105	−0.392 **	−0.190	−0.374 *	−0.411 **	1	
	*p*-value	0.465	0.077	0.246	0.004	0.131	0.036	0.001	-	
MPI	Rho	−0.160	−0.369 *	−0.214	−0.087	−0.262	−0.241	0.379 *	0.530 **	1
	*p*-value	0.327	0.012	0.794	0.672	0.149	0.350	0.020	0.001	-

** Correlation is significant at the 0.01 level (2-tailed); * correlation is significant at the 0.05 level (2-tailed); cTn—cardiac troponins; BNP—Brain natriuretic peptide, CK—creatine kinase; GLS—global longitudinal strain; SMOD—Simpson method of discs; MPI—myocardial performance index.

**Table 6:** Regression analysis for factors associated with cardiac toxicity.

Adjusted Factors *	Odds Ratio	(95% CI)	*p*-Value
Age	0.88	0.24–1.39	0.168
GLS	1.24	0.81–4.03	0.221
SMOD	4.05	1.33–7.40	<0.001
MPI	2.49	1.08–7.24	0.030
LVEF	3.16	2.13–9.66	<0.001
cTnI	1.41	1.35–2.70	0.042
cTnT	3.91	2.00–11.19	<0.001
BNP	1.22	0.94–1.63	0.094
NT-proBNP	2.15	1.38–6.05	<0.001
CK-MB	1.31	0.89–1.92	0.253
CK-MM	2.58	1.10–4.37	<0.001

*—The control group serves as reference; CI—Confidence Interval; GLS—global longitudinal strain; SMOD—Simpson method of discs; MPI—myocardial performance index; LVEF—left ventricle ejection fraction; cTn—cardiac troponins; BNP—brain natriuretic peptide; CK—creatin kinase.

## Data Availability

Data are available on request.
